# Syndecan-1 knock-down in decidualized human endometrial stromal cells leads to significant changes in cytokine and angiogenic factor expression patterns

**DOI:** 10.1186/1477-7827-8-133

**Published:** 2010-11-02

**Authors:** Dunja M Baston-Büst, Martin Götte, Wolfgang Janni, Jan-Steffen Krüssel, Alexandra P Hess

**Affiliations:** 1 University Düsseldorf, Medical Faculty, Department of OB/GYN and REI, Moorenstr. 5, 40225 Düsseldorf, Germany; 2 Department of OB/GYN, Münster University Hospital, Albert-Schweitzer-Str. 33, 48149 Münster, Germany

## Abstract

**Background:**

Successful embryonic implantation depends on a synchronized embryo-maternal dialogue. Chemokines, such as chemokine ligand 1 (CXCL1), play essential roles in the maternal reproductive tract leading to morphological changes during decidualization, mediating maternal acceptance towards the semi-allograft embryo and induction of angiogenesis. Chemokine binding to their classical G-protein coupled receptors is essentially supported by the syndecan (Sdc) family of heparan sulfate proteoglycans. The aim of this study was to identify the involvement of Sdc-1 at the embryo-maternal interface regarding changes of the chemokine and angiogenic profile of the decidua during the process of decidualization and implantation in human endometrium.

**Methods:**

A stable Sdc-1 knock-down was generated in the immortalized human endometrial stromal cell line St-T1 and was named KdS1. The ability of KdS1 to decidualize was proven by Insulin-like growth factor binding 1 (IGFBP1) and prolactin (PRL) confirmation on mRNA level before further experiments were carried out. Dot blot protein analyses of decidualized knock-down cells vs non-transfected controls were performed. In order to imitate embryonic implantation, decidualized KdS1 were then incubated with IL-1beta, an embryo secretion product, vs controls. Statistical analyses were performed applying the Student's t-test with p < 0.05, p < 0.02 and p < 0.01 and one way post-hoc ANOVA test with p < 0.05 as cut-offs for statistical significance.

**Results:**

The induction of the Sdc-1 knock-down revealed significant changes in cytokine and angiogenic factor expression profiles of dKdS1 vs decidualized controls. Incubation with embryonic IL-1beta altered the expression patterns of KdS1 chemokines and angiogenic factors towards inflammatory-associated molecules and factors involved in matrix regulation.

**Conclusions:**

Sdc-1 knock-down in human endometrial stroma cells led to fulminant changes regarding cytokine and angiogenic factor expression profiles upon decidualization and imitation of embryonic contact. Sdc-1 appears to play an important role as a co-receptor and storage factor for many cytokines and angiogenic factors during decidualization and implantation period, supporting proper implantation and angiogenesis by regulation of chemokine and angiogenic factor secretion in favour of the implanting embryo.

## Background

The successful establishment of a pregnancy in human depends on a synchronized dialogue of maternal and embryonic factors enabling attachment of the embryo to the uterine wall. Invasion into the decidualized maternal endometrial stroma is followed by induction of angiogenesis and acceptance of the semi-allograft embryo by the maternal immune system.

Cytokines are well-characterized factors in the implantation period [1]. Interleukin-1beta (IL-1β) was identified as a secretion product of human syncytiotrophoblast and trophoblast in early human pregnancy [2]. Furthermore, higher levels of IL-1β protein and chemokine ligand 1 (CXCL1) were shown to be expressed in the decidua of early pregnancy [3]. CXCL1 plays an important role in mediating the acceptance of the maternal immune system towards the semi-allograft embryo by attracting specialized leukocyte populations, such as uterine natural killer cells (uNK), granulocytes and macrophages supporting essential modifications regarding implantation and protection of pregnancy [3,4]. Chemokines belong to a subfamily of cytokines assigned by the position of conserved cysteins in their amino acid sequence. They function as chemoattractants for immunocompetent cells like leukocytes, which migrate towards the highest concentration of the chemoattractant, and act via G-protein coupled, 7 transmembrane-domains containing receptors on their target cells [5]. Especially CXCL1 was shown to be involved in early maternal reactions of the decidualized stroma to embryonic secretion products. This was depicted by a significant upregulation of *cxcl1 *gene expression after coincubation with trophoblast conditioned medium *in-vitro *as well as in a co-culture model of primary endometrial cells and trophoblast explants [6,7]. Besides the signalling through the classical G-protein coupled receptors, the heparan sulfate proteoglycan syndecans (Sdc) also take part as co-receptors in mediating chemokine function by enhancing the binding of chemokines to their innate receptors [8]. Sdcs are localized on the cell-surface and in the extracellular matrix. They consist of an ectodomain containing consensus sequences for heparan sulfate or chondroitin sulfate attachment, a single conserved transmembrane domain and a short cytoplasmic domain. Furthermore, they are described as multifunctional molecules in human, localized nearly ubiquitously and involved in wound healing, tumour growth, immune cell function and angiogenesis [9,10]. Lately, the mRNA expression of Sdc-1 to -4 in human endometrium of normal cycling healthy women was observed with a prominent up regulation of Sdc-1 and -4 in whole tissue secretory phase endometrium samples [11]. Sdc-1 was also found in uteroplacental units in human, localized apical in chorionic villi actively invading maternal decidua, supposedly being involved in embryo-maternal interaction [12].

We hypothesize that Sdc-1 plays an important role in the process of human decidualization and implantation by regulation of chemokine and angiogenic factor secretion of decidualized endometrial stroma cells supporting a proper embryonic attachment and subsequent implantation. In order to investigate this hypothesis, we generated a stable and inducible human endometrial stroma Sdc-1 knock-down cell line (called KdS1) which was further characterized on protein level by dot blot analysis regarding its cytokine and angiogenic factor expression profile. Furthermore, we investigated decidualized KdS1 (dKdS1) vs decidualized endometrial stroma cells (dSt-T1) after coincubation with the trophoblast secretion product IL-1β to identify Sdc-1's role and expression profile changes in the process of decidualization and implantation.

## Methods

### Cell line and cell culture

The human endometrial stroma cell line St-T1 used in this study was a generous gift from Professor Brosens (Imperial College, UK, Great Britain). These cells were initially isolated from normal proliferative endometrial tissue during diagnostic laparoscopy, immortalized, named St-T1 and characterized for functionality and comparability to primary endometrial stromal cells before [13,14]. They were maintained in a mixture of ¾ (v/v) DMEM and 1/4 (v/v) MCDB 105, supplemented with 10% (v/v) charcoal-stripped fetal bovine serum (FBS), 1× penicillin/streptomycin, 40 μg/ml gentamycin, 5 μg/ml insulin (Sigma-Aldrich, Steelze, Germany), 2 mM L-glutamine, 1 mM non-essential amino acids and 1× sodium pyruvate (all except insulin Biowest, Nuaillé, France).

### Transfection of pcDNA6/TR(c) via electroporation

**Figure 1:**
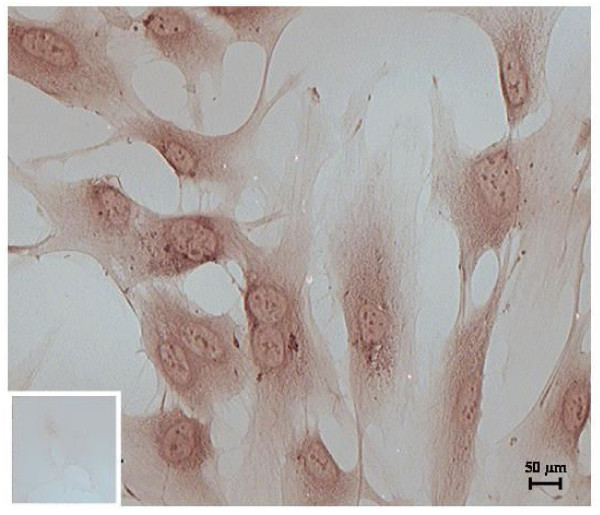
**Nuclear transfection of TetR in St-T1**. Representative immunocytochemic staining for nuclear TetR-expression in pcDNA6/TR(c) transfected St-T1 showing the clone with the most intense staining and negative control with non-specific IgG as small insert.

The TREx(tm)-system with the BLOCK-iT(tm) Inducible H1 RNAi Entry Vector Kit (Invitrogen, Karlsruhe, Germany) was chosen for stable, tetracycline (Tet) inducible Sdc-1 knock-down in immortalized St-T1. Therefore the plasmid pcDNA6/TR(c), coding for the Tet-repressor (TetR), was transfected first in order to generate a TetR stable expressing host cell line. One cell clone expressing high levels of TetR was chosen as host for the inducible knock-down of Sdc-1 regulating the expression of the short hairpin (sh) RNA of Sdc-1 in trans (Figure 1). The circular plasmid pcDNA6/TR(c) was transfected succesfully in St-T1 using Nucleofection(r) in a Nucleofector(r) I (Lonza Cologne AG, Cologne, Germany). 10^6^ cells were transfected per well with 3 μg plasmid DNA in buffer V (Lonza Cologne AG) using the following programs: T-13, -23, -24, U-17, -23 and -24. The selection with 5 μg/ml blasticidin started 48 h after transfection was performed.

### Immunocytochemistry for TetR

Transfected cells were tested for succesful nuclear transfection of TetR by immunostaining with mouse anti-TetR (500 μg/ml) (MoBiTec, Göttingen, Germany). Controls were stained with non-specific mouse immunoglobulin (IgG) (200 μg/ml) (Santa Cruz Biotechnologies, Santa Cruz, CA, USA). Cells were cultured with adequate antibiotics on Nunc(tm) Lab-Tek(r) glass chamber slides (Thermo Scientific Fisher, Langenselbold, Germany), fixed and permeabilized with cold acetone according to manufacturer's instructions for Vectastain(r) ABC-staining using an immunoperoxidase procedure (Vector Laboratories, Burlingame, CA, USA). Briefly, intracellular activity of peroxidases was quenched with 0.3% (v/v) H2O2 for 20 min and cells consecutively incubated with blocking serum for 1 h followed by incubation with 1^st^ antibody (1:200 in blocking serum) for 1 h. After incubation with the matching biotinylated 2^nd^ antibody, cells were incubated with ABC reagent and staining visualized by incubation with peroxidase substrate solution (DAB, Vector). Cells were washed, dehydrated, coverslipped and photographed with a Leica DC 300F microscope (Leica, Solms, Germany). The clone with the most intense staining for TetR was chosen for further transfection (Figure 1).

### Design of short hairpin RNAs

In a second step, short hairpin (sh) RNAs for the Sdc-1 knock-down were designed imitating tested transient silencer (si) RNAs for Sdc-1 (database sequence NM_002997) [15] using Invitrogen's RNAi Designer [16] following the manufacturer's instructions. The first four bases of the shRNA sequence are required for directed cloning in the vector pENTR(tm)/H1/TO (see Table 1).

**Table 1:** Design of Sdc-1 shRNAs

No.	sequence shRNA (5'3') (*loop sequence*)	database sequence NM_002997
**1**	**top **cac c**ag gac ttc acc ttt gaa acc ***cga a*gg ttt caa agg tga agt cc **bottom **aaa agg act tca cct ttg aaa cc*t tcg ***ggt ttc aaa ggt gaa gtc ct**	874**agg act tca cct ttg aaa cc**893
**2**	**top **cac c**ag gag gaa ttc tat gcc tga ***cga a*tc agg cat aga att cct cc **bottom **aaa agg agg aat tct atg cct ga*t tcg ***tca ggc ata gaa ttc ctc ct**	1162**agg agg aat tct atg cct ga**1181
**3**	**top **cac c**gg taa gtt aag taa gtt ga***c gaa *tca act tac tta act tac c **bottom **aaa agg taa gtt aag taa gtt ga*t tcg ***tca act tac tta act tac c**	1749**ggt aag tta agt aag ttg a**1767

Sequences of shRNAs versus Sdc-1 with siRNA sequences are bolded

### Cloning of shRNAs of interest in pENTR(tm)/H1/TO and sequencing

For ligation in the pENTR vector, double-strand (ds) oligo and vector were mixed in a molar ratio of 50:1 and ligation performed according to manufacturer's manual. Positive clones were analyzed by culturing 10 colonies each, followed by plasmid DNA isolation (GeneJET(tm) Plasmid Miniprep Kit; Fermentas, St. Leon-Rot, Germany) and sequencing with H1 forward and M13 reverse primers (Invitrogen) at the biomedical research center of the Heinrich-Heine University (Düsseldorf, Germany).

### Transfection of pENTR™/H1/TO cloned fragments in pcDNA6/TR© transfected St-T1, selection

Clones identified with correct inserted shRNAs for Sdc-1 - one from each pair (Table 1) - were transfected into the TetR expressing host cell line via Nucleofection(r) with program T-23 as described above. Selection with 200 μg/ml Zeocin(r) and 5 μg/ml Blasticidin started 48 h after Nucleofection(r).

### Primers for Polymerase Chain Reaction (PCR)

Sequences for β-actin, insulin-like growth factor binding protein-1 (IGFBP-1) and prolactin (PRL) mRNAs were obtained from the GenBank Database of the National Center for Biotechnology Information (NCBI) of the National Institutes of Health (NIH, [17]). Primers were synthesized by Eurofins MWG (Ebersberg, Germany). To ensure that the product detected resulted from amplification of cDNA rather than contaminating genomic DNA, primers were designed to cross intron/exon boundaries. Furthermore, PCR products were sequenced at the biomedical research center of the Heinrich-Heine University (BMFZ) (Düsseldorf, Germany) and confirmed. The sequences and the sizes of the amplified fragments are listed in Table 2.

**Table 2:** PCR and RT-PCR primers

	sequences 5' 3'	size of the amplified fragment [bp]
**β-actin**	**for **- cagggtgtgatggtgggaatgg **rev **- caggatggcgtgagggagagca	409
**IGFBP-1**	**for **- agtttagccaaggcacagga **rev **- tatctggcagttggggtctc	204
**PRL**	**for **- gcttctgtatcatctggtcacg **rev **- tgcgtaggcagtggagcag	247
**GAPDH**	**for **- tgcaccaactgcttagc **rev **- acagtcttctgggtggcagtg	131

Primers for amplification with PCR and RT-PCR

### RNA isolation, reverse transcription, PCR and real-time PCR

Total RNA was isolated from cells applying the single-step method described by Chomczynski & Sacchi [18] and processed as described before [19]. Prior to reverse transcription (RT), DNA-free RNA was generated by a desoxyribonuclease I (DNase I) (Fermentas, St. Leon-Rot, Germany) digestion [20] as described before [21]. RT reaction was performed using 2 μg RNA according to manufacturer's instruction for High Capacity cDNA archive kit (Applied Biosystems Inc, Foster City, CA, USA).

In subsequent PCRs, β-actin was used as a housekeeping gene and reactions consisted of 1× DreamTaq(tm) Green PCR Master Mix (Fermentas), 120 ng cDNA, 0.3 μM forward and reverse primer and dH2O ad 25 μl. After completion of 35 cycles of 94°C for 1 min, 94°C for 30 sec, 56°C (β-actin and IGFBP-1) and 53°C (PRL) for 45 sec and 72°C for 60 sec, the reaction was terminated at 72°C for 7 min and cooled down to 6°C in a peqSTAR 96 universal gradient thermocycler (PEQLAB Biotechnology, Erlangen, Germany). PCR-products were stored at -20°C until 2% agarose-gel electrophoresis was carried out in the presence of ethidiumbromide (0.2 μg/ml). After completion of electrophoresis, the agarose-gel was analyzed by the GelDoc 1000 system (Bio-Rad Laboratories, Hercules, CA, USA).

The semi-quantitative PCR (real-time PCR) for PRL and IGFBP-1 normalized to ß-actin and Glyceraldehyde-3-phosphate dehydrogenase (GAPDH) was performed using the Qiagen QuantiTect SYBR Green PCR kit in a LightCycler (Roche, Indianapolis, IN, USA) as described previously [22]. Amplification specificity was verified using melting curve analysis and 2% agarose gel electrophoresis of the PCR products. Primer sequences are listed in Table 2.

The semi-quantitative PCR for Sdc-1 was performed by collaborators using TaqMan technology (Applied Biosystems Inc). cDNA corresponding to 50 ng total RNA was used as a template in the PCR reaction consisting of ABI MasterMix (Applied Biosystems), and pre-designed TaqMan gene expression systems (Applied Biosystems) according to the manufacturers' instructions. For detection of Sdc-1, primer Hs00174579_m1 was used and normalized to the expression of mammalian 18S ribosomal RNA (rRNA) (Hs99999901_s1, all primers by Applied Biosystems). RT-PCR was performed using the ABI PRISM 7300 Sequence Detection System (Applied Biosystems) by using the default thermal cycling conditions (10 min at 95°C, and then 40 cycles of 15 sec at 95°C plus 1 min at 60°C). Data were analyzed using the comparative Ct (2^-ΔΔCt^) method [23] (3 biological and 2 technical repeats).

### Tet induction of Sdc-1 knock-down in double transfected St-T1

**Figure 2:**
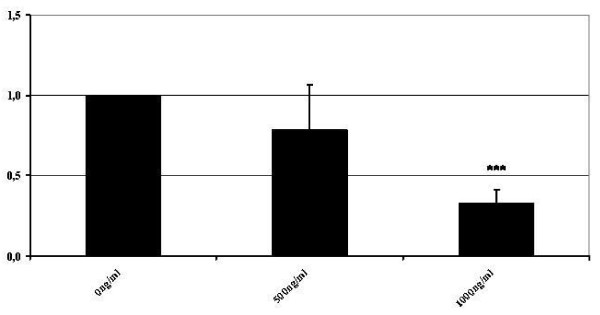
**Sdc-1 mRNA expression after induction with Tet**. Fold change of Sdc-1 mRNA after 48 h of induction with different Tet concentrations [0-1000 ng/ml] in KdS1 cells compared with 18S ribosomal RNA (***p < 0.01).

The clone resulting from pair 1 was tested for Tet-induction according to manufacturers' instructions from 0 to 1000 ng/ml Tet [0, 1, 50, 100, 500, 1000 ng/ml] for 24 and 48 hours and analyzed by RT-PCR for Sdc-1 mRNA expression. The mRNA expression decreased about 70% after 48 h with 1000 ng/ml Tet (Figure 2) (3 biological and 2 technical repeats). Subsequently, for further investigations an incubation time of 48 h with 1000 ng/ml Tet was used. The resulting clone was named knock-down Sdc-1 (KdS1).

### Subcellular protein fractionation and dot blot analysis for Sdc-1 expression

In order to examine knock-down of Sdc-1 on cellular level, KdS1 with and without incubation with Tet were analyzed for Sdc-1 expression via dot blot analysis after subcellular protein fractionation (3 biological repeats). Cells were processed following manufacturer's instruction for Subcellular Protein Fractionation Kit (Pierce Biotechnology, Rockford, IL, USA). 30 μg protein of each membrane fraction of KdS1 with and without Tet incubation was applied on methanol pre-treated polyvinylidene difluoride membranes (Immobilon(tm)-P PVDF Membranes, Millipore Corporation, Schwalbach, Germany). Human mononuclear cells isolated from peripheral blood served as control. Membranes were blocked with 5% fat-free milk powder (Sigma Aldrich), 0.1% Tween(r)20 (Sigma Aldrich) in phosphate-buffered saline (PBS) for 30 min, washed and incubated with mouse anti-human Sdc-1 antibody (1 μg/ml; abcam, Cambridge, UK) for 2 h. Finally, membranes were incubated with horseradish peroxidase conjugated ECL(tm) anti-mouse IgG (1 μg/ml; GE Healthcare, Buckinghamshire, UK), signals detected using RapidStep(tm) ECL Reagent (Calbiochem, Darmstadt, Germany) and analyzed with an Alpha Imager camera (Biozym Scientific GmbH, Hessisch Oldendorf, Germany) and the Alpha Ease FT 6.0.0. program (Alpha Innotech Corporation, San Leandro, CA, USA) (3 biological and 1 technical repeat).

### Decidualization of St-T1 and Tet-induced KdS1

St-T1 endometrial stroma cells (St-T1) are known to decidualize when treated with cyclic-AMP (0.5 mM) and progesterone (1 μM) for 72 h [13,14]. Double transfected and Tet-induced KdS1 cells were decidualized under the same conditions to investigate possible differences in their ability to decidualize due to the Sdc-1 knock-down. Decidualization was verified by carrying out a RT-PCR for the known decidualization markers IGFBP-1 and PRL.

### Investigation of chemokine and angiogenic factor profiles via dot blot analysis with cell culture supernatant of decidualized St-T1 and KdS1 with or without IL-1β incubation

dSt-T1 and dKdS1 (passages 3-6) were tested for IGFBP1 and PRL mRNA expression as described above. Cells were then incubated with or without IL-1ß (0.1 ng/ml) for 48 h according to previous investigations [24]. Cell supernatants were stored and applied to Proteome Profiler(tm) human chemokine and angiogenesis array kits (R&D Systems, Minneapolis, MN, USA) in order to identify the molecular changes caused by the Sdc-1 knock-down in human endometrial stromal cells (n = 4 each). Assays were performed according to manufacturers' instructions with 1 ml cell-culture supernatant containing 3×10^6^ cells per ml. Dot blots were photographed and analyzed with an Alpha Imager camera (Biozym Scientific GmbH) and the Alpha Ease FT 6.0.0. program (Alpha Innotech Corporation) (4 biological and 1 technical repeat).

### Statistical analysis

To investigate the statistical significance of the protein expressions, the Student's t-test with p < 0.05, p < 0.02 and p < 0.01 and one way post-hoc ANOVA test with p < 0.05 as cut-offs for statistical significance were carried out.

## Results

### Immunocytochemistry

The nuclear transfection of Tet(R) - coding plasmid pcDNA6/TR(r) was proven and evaluated via intensity rating of immunocytochemistry staining by two independent investigators (Figure 1). All six clones resulting from blasticidin selection were tested and the clone showing the strongest staining intensity for Tet(R) was selected for further transfection with Sdc1 shRNA.

### Tet-induction of Sdc-1 knock-down

The induction of Sdc1-RNAi in KdS1 with different Tet concentrations (0-1000 ng/ml) was tested for 24 and 48 h and Sdc1-mRNA was measured quantitatively by real-time PCR. A 70% reduction of Sdc1-mRNA was detected after incubation with 1000 ng/ml Tet for 48 h compared to untreated KdS1 (Figure 2). This concentration was used in the following experiments.

### Expression of Sdc-1 on cell membrane of KdS1

**Figure 3:**
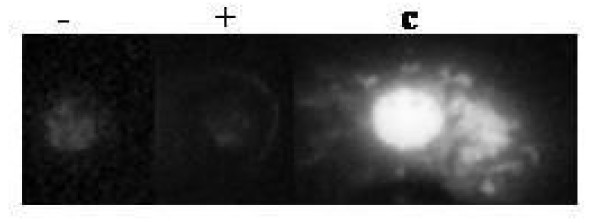
**Dot blot analysis of Sdc-1 expression in membrane fraction of KdS1**. Protein expression of Sdc-1 before (-) and after (+) induction with Tet in membrane fraction of KdS1. Mononuclear cells isolated from peripheral blood served as control (c).

Tet-incubated KdS1 cells were tested for the protein expression of Sdc-1 on cellular surface (KdS1 without Tet incubation served as controls). Human mononuclear cells served as positive controls. The expression of Sdc-1 in membrane compartment declined as shown in Figure 3.

### Decidualization

**Figure 4:**
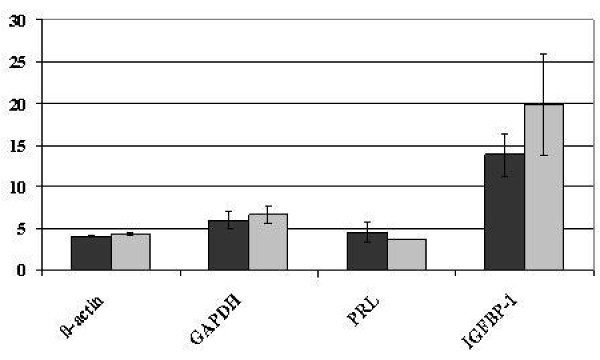
**PRL and IGFBP-1 mRNA expression in dKdS1 and dSt-T1**. Mean cycle threshold value (Ct) of PRL and IGFBP-1 mRNA expression compared with actin and GAPDH as internal standards after decidualization of KdS1 (dKdS1) (light grey bars) (dSt-T1 (dark grey bars) served as controls).

KdS1 cells were incubated with Tet for 48 h prior to 72 h of decidualization protocol and tested for the expression of common decidualization markers IFGBP1 and PRL. dSt-T1 served as controls. The mRNA expression of PRL and IGFBP-1 showed no statistical differences in dKdS1 versus dSt-T1 (Figure 4).

### Dot Blots after decidualization

**Figure 5:**
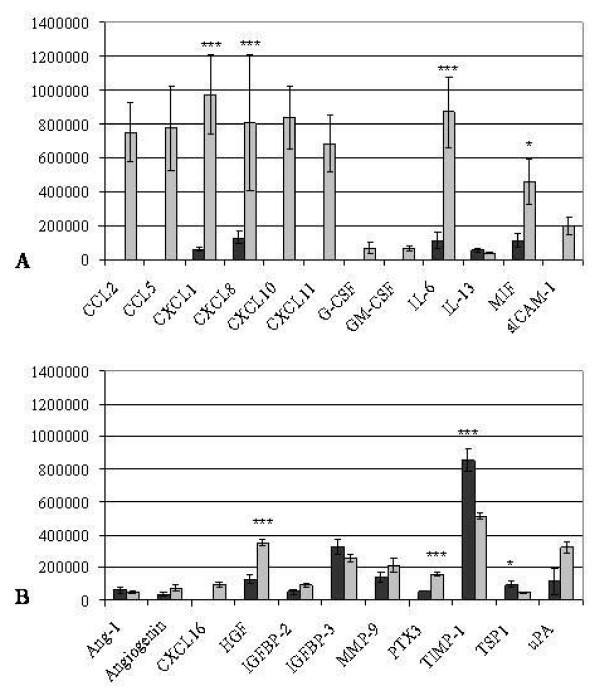
Pixel density analysis of cytokine (A) and angiogenic factor (B) dot blot array of decidualized dSt-T1 (dark grey bars) and dKdS1 (light grey bars) (n=4 each) (*p<0.05, **p<0.02, ***p<0.01). A) pixel analysis of secreted chemokines and B) pixel analysis of secreted angiogenic factors.

Dot blot arrays for cytokines (Figure 5A, Table 3) and angiogenic factors (Figure 5B, Table 4) were performed after 72 h decidualization of dKdS1 vs. dSt-T1 which served as controls.

**Table 3:** Cytokine profiling

cytokines	dSt-T1	dKdS1	dSt-T1+IL-1β	dKdS1+IL-1β
**CCL2**		**+**		**+**
**CCL5**		**+**		**+**
**CXCL1**	**+**	**+**	**+**	**+**
**CXCL8**	**+**	**+**	**+**	**+**
**CXCL10**		**+**		**+**
**CXCL11**		**+**		**+**
**G-CSF**		**+**		**+**
**GM-CSF**		**+**		**+**
**IL-6**	**+**	**+**	**+**	**+**
**IL-13**	**+**	**+**	**+**	
**MIF**	**+**	**+**	**+**	**+**
**sICAM-1**		**+**		**+**

Overview of cytokines in cell culture supernatant of decidualized endometrial stroma cells (dSt-T1), decidualized Sdc-1 knock-down endometrial stroma cells (dKdS1), decidualized endometrial stroma cells coincubated with IL-1β (dSt-T1 + IL-1ß) and decidualized Sdc-1 knock-down endometrial stroma cells coincubated with IL-1β (dKdS1 + IL-1ß). + indicates the presence, - the absence of the respective cytokine in the cell culture supernatant.

**Table 4:** Angiogenic factor profiling

angiogenic factors	dSt-T1	dKdS1	dSt-T1+IL-1β	dKdS1+IL-1β
**Ang-1**	**+**	**+**	**+**	
**Angiogenin**	**+**	**+**		**+**
**CXCL16**		**+**		**+**
**HGF**	**+**	**+**	**+**	**+**
**IGFBP-2**	**+**	**+**	**+**	**+**
**IGFBP-3**	**+**	**+**	**+**	**+**
**MMP-9**	**+**	**+**	**+**	**+**
**PTX3**	**+**	**+**	**+**	**+**
**TIMP-1**	**+**	**+**	**+**	**+**
**TSP-1**	**+**	**+**	**+**	**+**
**uPA**	**+**	**+**	**+**	**+**

Overview of angiogenic factors in cell culture supernatant of decidualized endometrial stroma cells (dSt-T1), decidualized Sdc-1 knock-down endometrial stroma cells (dKdS1), decidualized endometrial stroma cells coincubated with IL-1β (dSt-T1 + IL-1ß) and decidualized Sdc-1 knock-down endometrial stroma cells coincubated with IL-1β (dKdS1 + IL-1ß). + indicates the presence, - the absence of the respective angiogenic factor in the cell culture supernatant.

The chemokines CXCL1, CXCL8, IL-6 and macrophage migration inhibitory factor (MIF) were secreted at significantly higher levels in dKdS1. Comparable amounts of IL-13 were secreted from dKdS1 and dSt-T1.

The expression of several cytokines was restricted only to dKdS1. Secretion of CCL2, CCL5, CXCL10, CXCL11 and soluble intercellular adhesion molecule (sICAM-1) occurred in dKdS1 supernatant only. Furthermore, we detected the peptide hormone granulocyte colony-stimulating factor (G-CSF) and the glycoprotein granulocyte macrophage colony-stimulating factor (GM-CSF), both involved in immune cell differentiation and infection mechanisms, only in dKdS1 (Figure 5A).

When investigating angiogenic factors after decidualization, dKdS1 supernatant contained significantly more hepatocyte growth factor (HGF, scatter factor), a paracrine factor which stimulates mitogenesis, angiogenesis and tumorigenesis, and pentraxin 3 (PTX3), a soluble molecule which belongs to the innate immune system. On the other hand, secretion of tissue inhibitor of metalloproteinases 1 (TIMP-1) and thrombospondin 1 (TSP1), both components of the extracellular matrix, was significantly increased in dSt-T1.

The secretion levels of the angiogenic factors angiopoietin-1 (Ang-1), angiogenin, IGFBP-2 and -3, matrix metalloproteinase 9 (MMP 9) and urokinase-type plasminogen activator (uPA) were similar in dKdS1 versus controls. CXCL16 could be identified to be exclusively expressed in dKdS1 (Figure 5B). Results are shown in Table 3 and 4.

### Dot Blots after decidualization and IL-1β incubation

**Figure 6:**
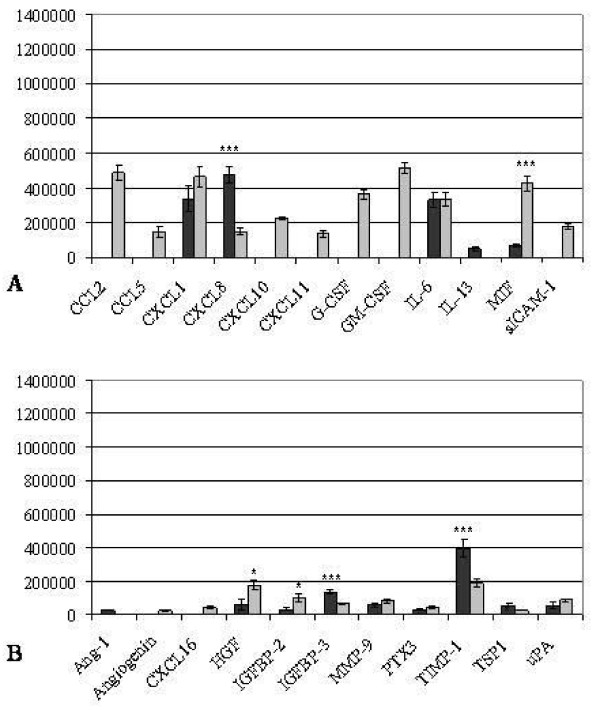
Pixel density analysis of cytokine (A) and angiogenic factor (B) dot blot array of decidualized dSt-T1 (dark grey bars) and dKdS1 (light grey bars) followed by an IL-1ß incubation for 48h (n=4 each) (*p<0.05, **p<0.02, ***p<0.01). A) pixel analysis of secreted chemokines and B) pixel analysis of secreted angiogenic factors.

Furthermore, dot blot arrays for cytokines (Figure 6A, Table 3) and angiogenic factors (Figure 6B, Table 4) were performed after 72 h decidualization followed by 48 h of coincubation with IL-1β imitating early embryonic contact in dKdS1 vs. the control dSt-T1.

Secretion of the cytokine MIF was significantly increased in dKdS1, whereas CXCL-8 secretion was higher in dSt-T1 cell culture supernatant. The secretion levels of CXCL1 and IL-6 were similar in dKdS1 and dSt-T1 after IL-1ß stimulation. Several cytokines associated with inflammatory mechanisms were only secreted in dKdS1 supernatant, namely G-CSF, GM-CSF, sICAM-1 and the chemokines CCL2, CCL5, CXCL10 and CXCL11. IL-13 was secreted in dSt-T1 exclusively (Figure 6A).

Obvious changes also occurred regarding the expression profile of angiogenic factors after embryo imitating IL-1ß contact. HGF and IGFBP-2 were secreted significantly higher in dKdS1 compared to dSt-T1. IGFBP-3, TIMP-1 and TSP1 dominated in dSt-T1 supernatant vs. dKdS1. There were no statistically significant differences for MMP9, PTX3 and uPA in dKdS1 vs. dSt-T1. Ang-1 was exclusively secreted from dSt-T1 and angiogenin and CXCL16 were restricted to dKdS1 (Figure 6B). Results are shown in Table 3 and 4.

## Discussion

The highly synchronized embryonic-maternal dialogue enables a proper implantation in human. In this study we have demonstrated an increase in chemokine and angiogenic factor secretion upon Sdc-1 knock-down in decidualized human endometrial stroma cells. The results are consistent with the role of Sdc-1 as a co-receptor and storage factor for these molecules allowing new insights in interactions and regulations between chemokines and angiogenic factors contributing to an improved understanding of intermolecular network at the embryo-maternal interface. Removal of Sdc-1 from endometrial stroma cell surfaces by the process of ectodomain shedding or down regulation of its expression leads to a down regulation of binding sites for chemokines and angiogenic factors. Recent studies suggested that Sdcs and especially Sdc-1 and-4 are involved in modifying the actions of chemokines and angiogenic factors by elongation of the ligand-receptor interactions, storage, establishment of a chemokine concentration gradient, shedding and regulation of growth-factor signalling cascades, respectively [9,25]. The recent observation that newborn Sdc-1 deficient mice are systemically smaller than their wild-type littermates suggests an important role for Sdc-1 during embryonic development [26]. Since animal models can only provide hints towards the human implantation period, human *in-vitro *cell-culture models may be more suitable to mimic the *in-vivo *situation of the human species. The immortalized, non-cancerogenous human endometrial stroma cell line St-T1 was demonstrated to function as an excellent model for human endometrium before [13,14]. Therefore, this model was used in this study.

The precise molecular actions of Sdc-1 underlying the morphological and structural changes during the menstrual cycle in preparation for embryo implantation or menstrual shedding in absence of fertilization are unknown until today. This study provides the first evidence of an important contribution of Sdc-1 in regulating chemokine and growth factor action during the decidualization and implantation processes.

Selected findings are discussed further in the following paragraphs.

### Chemokines in decidualization and early embryo-maternal dialogue

The glutamic acid - leucine - arginine (ELR) motif-positive chemokines CXCL1 and CXCL8 were secreted at higher levels in the Sdc-1 knock-down cells following decidualization, as compared to controls. Regarding secretion after contact with the embryo surrogate, IL-1ß, CXCL1 expression was comparable in both groups, whereas CXCL8 increased significantly in dSt-T1 supernatant compared to dKdS1. ELR-positive CXC-chemokines display angiogenic abilities, whereas chemokines lacking the ELR-motif are often characterized as angiostatic factors [27]. CXCL1 and CXCL8 bind to CXC-receptor 1 (CXCR1) and CXCR2, respectively [28,29]. Sdc-1 was shown to interact with CXCL1, CXCL8 and their receptors via its anionic heparan sulfate chain [30,31]. As Sdc-1 might be a storage molecule for chemokines, and has been shown to establish functional gradients for CXCL1 and CXCL8, the increase in CXCL1 and CXCL8 secretion in dKdS1 compared to normal dSt-T1 upon decidualization is most likely due to the absence of Sdc-1 in dKdS1 cells [30,31]. Furthermore, the rise of CXCL8 after embryo contact in normal decidualized cells links this molecule to early angiogenesis. Interestingly, CXCL8 remains low in dKdS1 after incubation with IL-1ß and nearly equal amounts of CXCL1 are secreted in dKdS1 and dSt-T1. This might be a hint for further chemokine intermolecular networks or a possible function of Sdc-1 in regulating the CXCL1 and CXCL8 signalling. Recent studies focused on Sdc-1's role in promoting tumour invasion in endometrial cancer cell lines *in-vitro *via nuclear factor-kappaB (NF-κB) signalling [32]. This signalling cascade was also shown to be involved in CXCL1 signalling in esophageal cancer and in CXCL8 secretion in primary human hepatocytes [33,34]. The reduced CXCL8 secretion in dKdS1 cells after IL-1ß contact might be based on a lack of NF-κB signalling or limited CXCL1 and CXCL8 synthesis in dKdS1.

C-C motif chemokine ligand 2 (CCL2, formerly monocyte chemotactic protein-1, MCP-1) was described to be secreted into the uterine lumen as well as in the endometrial stroma by human primary endometrial epithelial cells and in first trimester decidua tissue functioning as a key player of monocyte chemotaxis [35,36]. Furthermore, CCL2 and its receptor CCR2 were detected in human first trimester decidual tissues with CCL2 being constantly secreted by decidual stromal cells via extracellular signal-regulated kinase (ERK) and mitogen-activated protein kinase (MAPK) signalling [37]. Since we detected CCL2 only in dKdS1 supernatant upon decidualization and imitation of embryo contact, we propose an active role for Sdc-1 in binding of CCL2 in human decidualized endometrium *in-vitro*. This storage of CCL2 by Sdc-1 in dES *in-vitro *seems to be inconsistent with the *in-vivo *situation of early pregnancy described in the literature [37]. Hence, there might be a temporal regulation of CCL2 expression as these data reflect the very early embryo-maternal dialogue. The absence of CCL2 expression in dES *in-vitro *might result from the absence of factors that influence the decidua *in-vivo*.

Two further CXC-motif chemokines - CXCL10 and 11 - were only secreted in dKdS1 with and without IL-1ß coincubation. The secretion of CXCL10 and 11 might result from the lack of Sdc-1 in dKdS1 cell membranes. In dSt-T1, these molecules are supposingly stored at the cell membrane via Sdc-1 inhibiting early secretion in the supernatant. Therefore, the secretion of these factors in the environment might lead to a shift in the early immune response of the endometrium and a misdirected angiogenesis, respectively.

Another chemokine, macrophage migration inhibitory factor (MIF), was significantly more secreted in dKdS1 after decidualization as well as after incubation with IL-1ß compared to controls. MIF binds to G-protein coupled receptors CXCR2 and CXCR4 and is involved in monocyte and T-cell chemotaxis as well as in activation of integrins and calcium influx [38]. In human endometrium, an increase in MIF expression was found in the late proliferative phase followed by a decline during the window of implantation suggesting different roles for MIF during the menstrual cycle [39]. In our opinion, MIF takes part in the endometrial decidualization process supported by the presence of MIF in decidualized endometrial cells (dSt-T1) where its secretion seems to be regulated by Sdc-1. dKdS1 lack this regulation of MIF and therefore MIF is more secreted after Sdc-1 knock-down. This regulation might also be initiated by NF-κB signalling similar to the findings in human primary endometrial cells [40].

### Angiogenic and growth factors in decidualization and early embryo-maternal dialogue

The process of endometrial angiogenesis already starts in the proliferative phase of the human menstrual cycle and peaks in the secretory phase. A synchronized pattern of angiogenic factors, growth factors and their inhibitors is therefore a critical precondition supporting not only angiogenesis but also modification of the extracellular matrix, decidualization and re-organization of the endometrial stroma.

The paracrine and angiogenic active, glycosaminoglycan-binding hepatocyte growth factor (HGF) was characterized as an essential key player of trophoblast invasion mediating trophoblast growth and differentiation via the signal transducers and activators of transcription (STAT3) signalling pathway after binding to the receptor c-mesenchymal-epithelial transition factor (c-Met) localized on cytotrophoblast [41-43]. Furthermore, HGF takes part in embryonic mesenchymal-endothelial interactions and organogenesis as well as in endometrial stromal cell invasion [44,45]. Sdc-1 has been assigned as a HGF co-receptor intensifying HGFs signalling via c-Met [46]. The significant increase of HGF in dKdS1 supernatant after decidualization and after IL-1ß contact as seen in this study, suggests an intense interaction between Sdc-1 and HGF. It seems that Sdc-1 is important in HGF-binding and presentation towards its receptor for supporting cell proliferation and angiogenesis during the implantation period. Due to Sdc-1 knock-down HGF-depending processes might be dysregulated.

Furthermore, we were able to show that the extracellular matrix glycoprotein thrombospondin 1 (TSP1) was significantly more secreted by normal decidualized and IL-1β incubated dSt-T1 compared to dKdS1. Previous studies focussed on its involvement in endometrial vascularization and decidualization mediated by interferon γ. A modified maternal immune response due to a reduced TSP1 expression of decidual macrophages was described in women with unexplained recurrent embryonic miscarriage [47]. Sdc-1 was found to interact with TSP1 in the formation of stable cellular matrix contacts via fascin spikes [48]. We suggest that the lack of TSP1 secretion in dKdS1 is based on the absence of the known NF-κB activation by IL-1β in dKdS1. Therefore, the absence of TSP1 might lead to angiogenic malformation and dysregulation of the decidualized endometrium possibly leading to implantation failure.

The progesterone-dominated secretory phase endometrium undergoes dramatic changes in matrix reconstruction and differentiation depending on an orchestrated pattern of proteases and inhibitors. Matrix metalloproteinases (MMPs) play an important role during the implantation phase regulated by their inhibitors, e.g. tissue inhibitor of metalloproteinase 1 (TIMP1), localized in embryonic and maternal tissues [49,50]. TIMP1 is the most secreted factor in dSt-T1 after decidualization and IL-1β stimulation. The knock-down of Sdc-1 in dKdS1 therefore leads to a dysregulation of the TIMP-1 expression possibly enabling an unlimited invasion. Former studies showed an involvement of Sdc-1 in MMP-9 regulation, a main target of TIMP-1, mediating endometrial cancer invasion [32]. These conclusions are supported by a decreased secretion of TIMP-1 in MCF-7 breast cancer cells overexpressing soluble Sdc-1 ectodomain, supporting breast cancer invasiveness [51]. A decreased expression of TIMP-1 might result in a dysbalanced implantation as similar levels of MMP-9 were shown in this study possibly leading to an overinvasion since the restain mechanism via TIMP-1 is disordered.

The secretion of long pentraxin 3 (PTX3) was most significantly elevated in dKdS1 supernatant upon decidualization. PTX 3 is a factor of the innate immune system and is expressed by a huge variety of cells, including macrophages, endothelial cells, fibroblasts and monocytes [52]. Recent studies report a distinct role for PTX3 mediating decidualization and fertilization in mice [53]. The increase in PTX3 in dKdS1 supernatant upon decidualization might result from a stronger inflammatory response in dKdS1 as indicated by the elevated secretion levels of other chemokines, e.g. CXCL8, compared to dSt-T1. Moreover, a possible dysregulation of components of the extracellular matrix caused by the Sdc-1 knock-down might activate the PTX3 release in order to induce apoptosis, as it was reported for human neutrophils [54].

### Infection-associated molecules in decidualization and early embryo-maternal dialogue

The modulation of the maternal immune system enabling an embryonic invasion is one of the key processes of the early fetal-maternal communication. The important role of cytokines, like IL-1ß system, influencing decidualization- and implantation-related molecules, like MMPs, has already been reported [55]. A recent study examined the components of endometrial secretions aspirated prior to embryo transfer in IVF and ICSI cycles, revealing the presence of pro-, as well as anti-inflammatory immuno-associated molecules [56]. The pro-inflammatory cytokine IL-6 belonged to the main secretion products. In the present study, IL-6 secretion significantly increased in dKdS1 after decidualization. The increase in IL-6 secretion might be due to the lack of Sdc-1 and displays a potential intermolecular interaction between IL-6 and Sdc-1. These data correlate well with observations in Sdc-1 deficient mice subjected to kidney and allergic inflammation where an increase of IL-6 was observed [57]. Hence, the lack of Sdc-1 might induce an inflammatory response in dKdS1 upon decidualization. The similar expression of IL-6 in both groups upon IL-1ß incubation might result from a balancing effect of other factors that are activated upon IL-1ß contact in dKdS1.

The cytokines granulocyte (G-) and granulocyte-macrophage (GM-) colony stimulating factors (CSF) are involved in the implantation period and early fetal maternal dialogue. The interaction between G-CSF and heparan sulfate has been shown in human long-term culture-initiating cells being raised upon a stromal feeder layer [58]. Herein, we detected G-CSF as well as GM-CSF secretion only in dKdS1 with an increase after IL-1ß incubation which underscores a very likely interaction of these colony-stimulating factors with Sdc-1.

## Conclusions

The present study therefore underscores the importance of Sdc-1 as a co-receptor and a storage molecule in the receptive endometrium supporting a proper biochemical basis for embryonic implantation. Further investigation of Sdc-1s role in the process of implantation is needed, since its synchronous regulation of multiple receptor-dependent pathways seems to be a pivotal point in understanding and possibly ameliorating human implantation.

## List of abbreviations

Ang: angiopoietin; CCL/CXCL: chemokine ligand; c-Met/HGFR: c-mesenchymal-epithelial transition factor/HGF receptor; CXCR: chemokine ligand receptor; d: decidualized; ELR: glutamic acid - leucine - arginine; ERK: extracellular signal-regulated kinase; G-/GM-CSF: granulocyte-/granulocyte-macrophage colony stimulating factor; HGF: hepatocyte growth factor; IGFBP: insulin-like growth factor binding protein; IL: interleukin; KdS1: endometrial Sdc-1 knock-down cell line; LH: luteinizing hormone; MAPK: mitogen-activated protein kinase; MIF: macrophage migration inhibitory factor; MMP: matrix metalloproteinase; NF-κB: nuclear factor kappa B; PTX3: pentraxin 3; Sdc: Syndecan; shRNA: short hairpin RNA; sICAM: soluble intercellular adhesion molecule; St-T1: immortalized endometrial stroma cell line; VEGF: vascular endothelial growth factor; uNK: uterine natural killer cells; Tet: tetracycline; TIMP: tissue inhibitor of metalloproteinases; TSP: thrombospondin; uPA: urokinase-type plasminogen activator

## Competing interests

The authors declare that they have no competing interests.

## Authors' contributions

DMBB performed the experimental outline and design of short hairpin RNAs, performed cell culture and decidualization, primer design for RT-PCR and the establishment of the knock-down cell line KdS1 Furthermore, DMBB performed the isolation of RNA and dot blot analysis. DMBB was responsible for the interpretation of the data and the writing of the manuscript. MG participated in knowledge transfer regarding Syndecan-1, performed *real-time *PCR and helped with the interpretation of the data. WJ helped to draft the manuscript. JSK participated in the statistical analysis of the data and edited the manuscript. APH generated the idea of the study, participated in the experimental outline and performed immunocytochemistry. APH participated in the interpretation of the data and the design of the manuscript. All authors read and approved the final manuscript.
